# Investigation of the Mechanical Properties of a Ceramic Material Fabricated Using Additive Manufacturing Technology

**DOI:** 10.3390/ma18225165

**Published:** 2025-11-13

**Authors:** Arkadiusz Popławski

**Affiliations:** Faculty of Mechanical Engineering, Military University of Technology, Kaliskiego 2 St., 00-908 Warsaw, Poland; arkadiusz.poplawski@wat.edu.pl

**Keywords:** ceramic additive manufacturing, stereolithography (SLA), sintering parameters, porosity and microstructure, mechanical properties

## Abstract

Additive manufacturing (AM) of ceramics has rapidly evolved over the past decade, enabling the production of complex, high-precision components with tailored porosity and geometry. Among AM techniques, stereolithography (SLA) and digital light processing (DLP) are particularly promising for fabricating dense and functional oxide ceramics. However, the final properties of printed ceramics are strongly affected by sintering conditions, layer geometry, and microstructural uniformity. This study presents a two-stage experimental approach to evaluate the influence of sample geometry, layer thickness, and sintering schedule on the mechanical and microstructural performance of SLA-printed ceramic parts. In Stage I, the relationships between elastic modulus (E_c_) and compressive strength (σ_c_) were examined as a function of sample height, layer thickness (0.05 and 0.10 mm), and firing program. In Stage II, the effects of sintering temperature (1250, 1271, and 1300 °C) and holding time (2–20 min) were analyzed for the reference geometry. Microstructural characterization, including pore size distribution and quantitative porosity analysis, was conducted to establish correlations with the mechanical results (Stage III). The findings reveal that optimized sintering and geometry parameters can significantly enhance mechanical performance and reduce porosity variations. The study provides both scientific insights and engineering guidelines for improving the structural reliability of SLA-fabricated ceramic components.

## 1. Introduction

Additive methods, including ceramic 3D printing, have undergone dynamic development over the past decade, finding applications in industries that simultaneously require high-dimensional precision, geometric accuracy, and stable performance under mechanical and thermochemical loads [1,2]. Travitzky et al. [3] provided a detailed description of various ceramic printing technologies. In the case of direct selective laser sintering (direct SLS), it is difficult to achieve a precise surface finish of printed components while maintaining a relatively low density, reaching only about 56% of the theoretical value. In the indirect SLS process, the achievable density is approximately 36% of the theoretical density, which can be increased up to 92% when the process is followed by hot isostatic pressing (HIP). Another method, ceramic stereolithography (CSL), enables the fabrication of hydroxyapatite implants with porosity in the range of 35–75% and compressive strength of about 30 MPa. This material is frequently used in medical applications as a scaffold for bone tissue regeneration.

Stereolithography (SLA) is currently one of the most commonly used additive manufacturing methods for ceramics, combining high printing resolution with surface smoothness and dimensional repeatability [4,5,6]. In this process, a photocurable resin contains a high ceramic powder loading (typically 45–55 vol.%), and the fabricated “green parts” require controlled debinding (removal of the organic phase) followed by sintering at a temperature suitable for the specific ceramic system [7]. The literature emphasizes the crucial influence of particle fraction and morphology, suspension viscosity and rheology, exposure parameters, layer thickness, and the thermal program (heating rate and isothermal dwell stages) on the final porosity, shrinkage, and mechanical properties of the material [8,9].

Doreau et al. [10] demonstrated that it is possible to fabricate ceramic components (Al_2_O_3_, ZrO_2_) using the SLA method with dense photopolymer pastes, achieving density and mechanical properties comparable to those of conventionally sintered ceramics. These pastes consisted of ceramic powders (Al_2_O_3_ or ZrO_2_) dispersed in a photocurable acrylic resin together with a photoinitiator, thickener, and dispersant. The solid loading in these pastes reached up to 67 vol.%, enabling the fabrication of homogeneous green bodies with densities comparable to conventionally sintered ceramics. This work initiated the development of modern CSL (Ceramic Stereolithography) and LCM (Lithography-based Ceramic Manufacturing) techniques, which are now widely used in research on technical ceramics and biomaterials.

Technical ceramics, particularly those based on silica, aluminosilicates, and zirconia, are valued for their high hardness, wear resistance, low thermal expansion coefficient, and chemical inertness. Piconi and Maccauro [11] described the phase stability of Y-TZP zirconia and its resistance to aging under biological conditions, which makes it a reference material for medical applications. Prints made from this material require precise slurry preparation and strict control of the sintering process to achieve the desired microstructure. Deville [12] presented a method for fabricating ceramics that enables control over porosity and pore orientation within the microstructure. The pore morphology depends mainly on the type of solvent, freezing rate, and temperature gradient, and the resulting material exhibits high anisotropy and a favorable strength-to-porosity ratio.

Conventional forming techniques, such as pressing, gel casting, or injection molding, limit the design freedom of complex geometries and the ability to control the microstructure of thin-walled components. These limitations can be overcome through layer-by-layer 3D printing, in which the geometry of the part no longer poses a technological constraint. Deckers et al. [13] observed that industrial ceramic printing is becoming increasingly cost-effective, and with proper optimization of process parameters and, if necessary, densification of the green parts, it is possible to obtain components free from cracks and pores. The combination of colloidal techniques with additive manufacturing further improves the homogeneity and density of the material.

Zocca et al. [14] emphasized that additive manufacturing methods enable the production of ceramic components with diverse shapes, structures, and controlled porosity, which are difficult to achieve using conventional techniques. Moreover, the sintering process is a crucial stage in obtaining the final components, as it significantly affects the ceramic structure (porosity) and, consequently, the physical and mechanical properties as well as the geometry of the parts [15,16,17,18]. Typical temperature ranges reported in the literature include the debinding stage, occurring between 500 and 700 °C, and the main sintering stage, carried out at approximately 1100–1450 °C for oxide ceramics (ZrO_2_, Al_2_O_3_), and up to 1600–1750 °C for non-oxide ceramics such as Si_3_N_4_.

Chen et al. [19] demonstrated in a comprehensive review that 3D-printed ceramics can exhibit properties comparable to those of highly sintered materials, provided that the paste composition is homogeneous and proper dehydration is ensured when using Digital Light Processing (DLP) technology. The authors emphasized that the sintering process is a critical stage for achieving optimal final properties, as even small temperature variations can significantly improve or degrade the microstructure. Increasing the solid-phase content in the particle carrier (paste or resin) leads to a reduction in shrinkage and an increase in the final density of the product. According to their analysis, slurry-based systems used in stereolithography or DLP typically contain between 30 and 65 vol.% of ceramic particles, where higher solid loading results in reduced shrinkage, higher density, and improved mechanical performance.

Zhang et al. [20] analyzed the influence of atmosphere and heating rate during debinding on the microstructure and defects of ceramic components fabricated using the DLP method, showing that higher heating rates promote the formation of more defects, such as cracks and delamination. Ramli et al. [21] demonstrated that increasing the sintering temperature reduces the number of pores but increases their average size due to intensified diffusion processes. De Jonghe et al. [22] noted that local differences in the rates of binder removal and densification lead to the development of internal stresses, which promote microcracking and heterogeneity in porosity. Similarly, German [23] reported that differences in interlayer diffusion and component thickness can result in the accumulation of structural defects. Insufficient sintering temperatures lead to incomplete densification, whereas excessively high temperatures cause melting of glassy phases, stress relaxation, and a reduction in load-bearing capacity [24].

In ceramic materials, quantitative analysis of porosity and pore distribution is particularly important. Microstructural studies indicate that pore size distributions vary depending on the ceramic composition and processing conditions [25]. Lin et al. [26] demonstrated that the size, distribution, and shape of pores play a crucial role in thermal conductivity, as smaller pores effectively limit heat transfer by increasing the contribution of the solid-phase surface area. Wu et al. [27] developed a method for producing porous mullite ceramics using 3D printing and polymethyl methacrylate (PMMA) templates, which enables control of pore distribution. Microscopic analysis confirmed the relationship between the size of the pore-forming particles and the uniformity of the structure. The mechanical and thermal properties of the material are strongly correlated with porosity and pore distribution, highlighting the importance of precise microstructural control in the design of technical ceramics.

For this reason, in microstructural analysis, it is important to present not only the total porosity but also the number-weighted and area-weighted pore size distributions, which make it possible to identify the dominant pore fractions contributing to the overall porosity [28]. It should be noted that measurements of porosity and pore distribution, whether performed using optical microscopy or X-ray tomography, are subject to uncertainty resulting from image segmentation, threshold selection, and preprocessing filters. Jaques et al. [29] indicated that differences in segmentation thresholds can change the calculated porosity by several percent. Kokhan et al. [30], Papia et al. [31], and Varoto et al. [32] confirmed that the choice of thresholding method, whether classical or based on machine learning, has a significant effect on the resulting pore size distribution and the detection of small defects. Kazup and Gácsi [33] pointed out that the scanning resolution and the quality of microscopic images largely determine the measurement accuracy and that results obtained using different methods (image analysis, µCT, porosimetry) may differ by several percentage points.

Despite the large number of publications on ceramic 3D printing [34], there are still few consistent and systematic studies that correlate the geometry of the printed element (height, diameter, layer thickness) and the burnout procedure with the sintering process and the results of mechanical tests, such as compression tests, particularly for commercial resins. Typically, only the manufacturers’ data specifying the parameters required to obtain defect-free prints are available [35,36,37].

Considering the above, the aim of this study is to provide a comprehensive evaluation of the influence of sample geometry, printing layer thickness, burnout procedure, and sintering parameters on the mechanical properties of ceramics fabricated by the SLA method using a ceramic resin. The research was conducted in two stages. In the first stage, the relationship between the compressive modulus (E_c_) and compressive strength (σ_c_) and the sample height, layer thickness (0.05 and 0.10 mm), and the burnout method was analyzed. In the second stage, the effect of sintering temperature (1250, 1271, and 1300 °C) and holding time (2–20 min) on the same mechanical properties was investigated for the reference geometry. For representative cross-sections, a microstructural analysis was performed to determine the pore size distribution and the contribution of individual fractions to total porosity, which made it possible to correlate microstructure with mechanical properties. The data processing methodology included statistical analysis (mean values, standard deviations, and confidence intervals based on Student’s t-distribution) and a consistent graphical presentation. Detailed numerical datasets are provided in the Appendix A.

This study has both cognitive and application-oriented aspects. From a scientific perspective, it presents data that distinguish the effects of printing geometry and burnout schedule from those of sintering temperature and time, while simultaneously linking them with porosity and pore size distribution. From an engineering standpoint, a properly designed ceramic burnout process can lead to an increase in compressive strength (the parameter with lower variability) or to achieving a balanced stiffness and good repeatability, which is particularly important in the design of thin-walled functional components fabricated using SLA technology.

## 2. Materials and Methods

### 2.1. Material Characterization

Ceramic samples (SiO_2_) were fabricated using additive manufacturing with the SLA technology on a Formlabs Form 2 printer (Formlabs, Somerville, MA, USA). The ceramic resin used in this study is an experimental material that enables the production of components that, after an appropriate burnout process, are transformed into a fully ceramic material with high-dimensional stability and thermal resistance. The resulting sintered ceramics exhibit good electrical insulation, a low coefficient of thermal expansion, and chemical resistance to most reagents.

**Figure 1:**
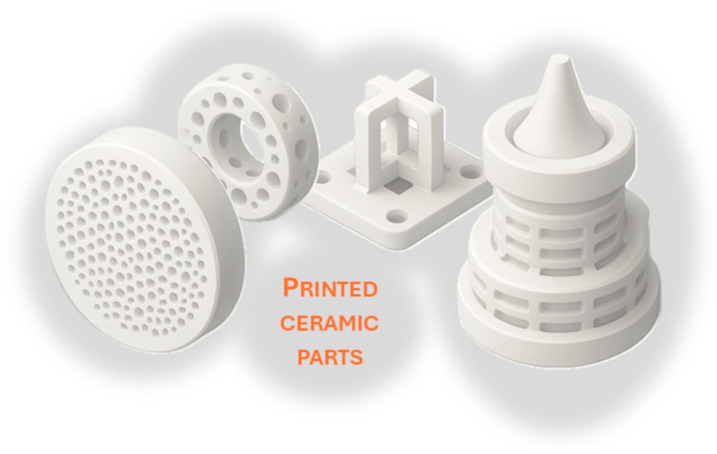
Example of ceramic parts that can be produced by stereolithography (SLA) using ceramic resin, inspired by Formlabs materials [35].

These properties make the material suitable for components operating at elevated temperatures, such as protective housings, insulators, supports in laboratory furnaces, and structural parts exposed to aggressive environments. However, due to their limited mechanical strength and relatively high porosity, printed silica ceramics are not intended for dynamically loaded applications. Their potential lies primarily in the fabrication of engineering prototypes, research models, and components with complex geometries that are difficult to produce using conventional ceramic processing methods (Figure 1). The precision of SLA technology allows for the manufacturing of thin-walled structures, porous elements, and artistic details with high surface quality.

According to the manufacturer, the ceramic material exhibits significant differences in mechanical and thermal properties between the state after printing (“green state”) and the state after firing (“fired state”).

In the green state, that is, immediately after the printing process and laser curing, the material behaves as a photocurable composite with fine ceramic filler particles (average particle size of 2 µm [9]). In this condition, it shows limited mechanical strength, with a tensile strength of 5.1 MPa, an elastic modulus of 1 GPa, and an elongation at break of 1.4%. The flexural properties are also low, with a fracture stress of 10.3 MPa and a flexural modulus of 995 MPa. The material remains prone to brittle fracture but is sufficiently stable to allow handling and preparation for the burnout process.

After firing at the standard temperature of approximately 1275 °C, the material undergoes sintering and becomes fully ceramic. In this state, the properties associated with the polymer phase disappear, and the structure acquires the characteristics typical of silica ceramics. High stiffness (elastic modulus 5.1 GPa), excellent dimensional stability, and thermal resistance. The fired material retains a similar flexural strength (10.3 MPa) but exhibits significantly improved thermal performance, with a heat deflection temperature of 75 °C at 1.8 MPa and above 290 °C at 0.45 MPa [35]. According to previous experimental results reported by the author [9], the green (unsintered) parts printed from Formlabs Ceramic Resin (Formlabs, Somerville, MA, USA) exhibited a true density of 1.72 g/cm^3^, corresponding to an estimated solid loading of approximately 38 vol.% of silica in the photopolymer matrix. After sintering at 1271 °C, the material reached a true density of 2.31 g/cm^3^, which is about 87% of the theoretical density of pure silica (2.65 g/cm^3^). The chemical composition determined by SEM–EDS was Si—36.9 wt.%, O—50.1 wt.%, Al—6.6 wt.%, Na—4.1 wt.%, and K—2.4 wt.%, confirming the silica-based character of the ceramic matrix.

### 2.2. Specimen Preparation

**Figure 2:**
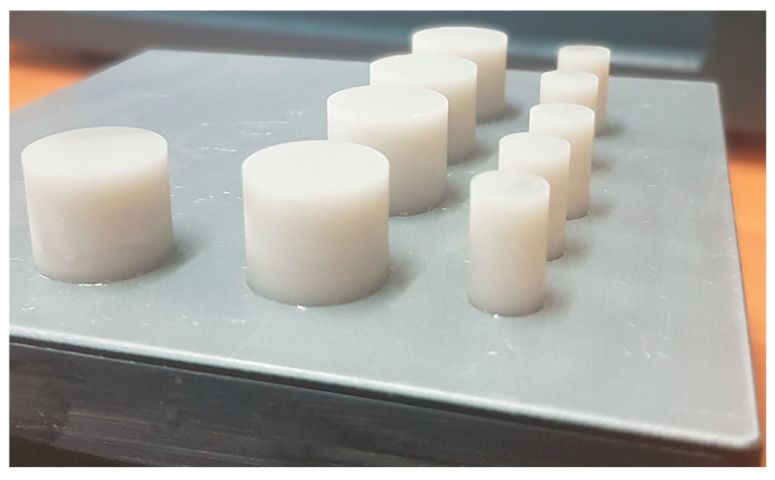
Samples printed from polymer resin directly on the build platform.

All specimens used in the study had a cylindrical shape with varying dimensions depending on the stage of the experiment (Figure 2). The exact dimensions are provided in the description of the experimental procedure. The samples were fabricated using the previously mentioned Formlabs Form 2 printer, employing the manufacturer’s recommended parameters as listed below:

- Layer thickness: 100 μm and 50 μm,
- Laser spot diameter: 140 μm,
- Exposure time per layer: approximately 15 s,
- Printing speed: approximately 50 mm/h (depending on the model geometry),
- Working chamber temperature: approximately 30–35 °C.

The specimens were fabricated directly on the printer’s build platform (Figure 2). They were then rinsed with isopropyl alcohol for 5 min to remove any residual uncured resin and to prevent structural damage to the material. Unlike other materials used in SLA technology, the ceramic resin does not require additional UV post-curing. For this reason, the rinsed specimens were only dried before proceeding to the subsequent processing steps.

After drying, the specimens were subjected to a burnout process in a Magma Therm MT-1300-5-B2 laboratory chamber furnace (Danlab, Białystok, Poland) equipped with an electronic temperature controller and a chamber ventilation system. The furnaces allow precise time–temperature programs to be carried out with an accuracy of ±1 °C and a maximum operating temperature of up to 1300 °C.

**Figure 3:**
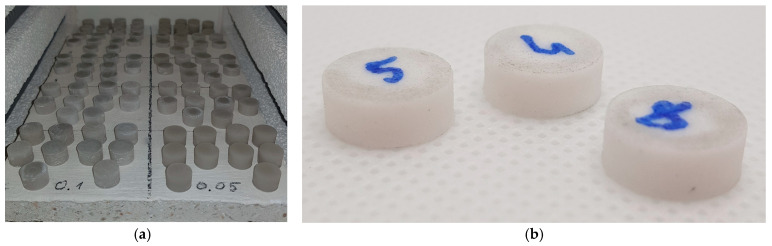
Specimens used in the study: (**a**) samples inside the furnace chamber immediately after firing; (**b**) representative samples removed from the furnace after firing, with sample numbers indicated.

Figure 3a shows the appearance of the ceramic specimens inside the furnace chamber after the completion of the sintering process.

After firing, the specimens acquired a light gray, uniform color with a characteristic matte surface sheen typical of silica ceramics (Figure 3b). Some samples exhibited slight translucency at the edges, indicating a properly executed sintering process. No surface deformation or cracking was observed, confirming the correctness of the adopted burnout program. The specimen dimensions decreased by approximately 14–20%, in accordance with the data provided by the manufacturer of the Formlabs Ceramic Resin.

### 2.3. Research Program

The research program was designed to provide a comprehensive analysis of the influence of selected technological parameters of the manufacturing and firing processes on the mechanical properties and structure of ceramics produced by the SLA additive manufacturing method using Formlabs Ceramic Resin. The study consisted of two stages, each focusing on a different aspect of the technological process, namely sample geometry and sintering parameters.

In the first stage (Stage I), the influence of geometry and printing parameters on the quality and mechanical strength of the ceramic material after sintering was analyzed. Cylindrical samples with a constant diameter of 10 mm and variable height ranging from 3 to 7 mm were printed using two different layer thicknesses (0.05 and 0.10 mm). Additionally, two burnout variants of the polymer matrix were considered: one with a constant time and another with a time proportional to the sample height. The purpose of this stage was to determine how the shape and thermal process parameters affect the burnout efficiency, the risk of defect formation, and the resulting compressive strength.

In the second stage (Stage II), the study focused on the influence of sintering parameters on the mechanical properties of the geometry identified in Stage I as having the highest compressive strength (sample with a height of 4 mm printed with a layer thickness of 0.10 mm). In this stage, samples were sintered at different temperatures (1250, 1271, and 1300 °C) and with various holding times at the maximum temperature (2, 5, 10, 15, and 20 min). The aim of this stage was to determine the effect of these parameters on the degree of densification, porosity, microstructure, and resulting mechanical strength of the ceramic sinter. A summary of the research program is presented in Table 1.

Conducting the research in two stages made it possible to systematically evaluate the influence of geometry, printing parameters, and thermal conditions on the properties of additively manufactured ceramics. The results of these analyses will serve as the basis for developing technological guidelines for the printing and firing processes, enabling the achievement of optimal density, structural uniformity, and high repeatability of the mechanical properties of printed ceramics.

### 2.4. Measurement Methods

**Figure 4:**
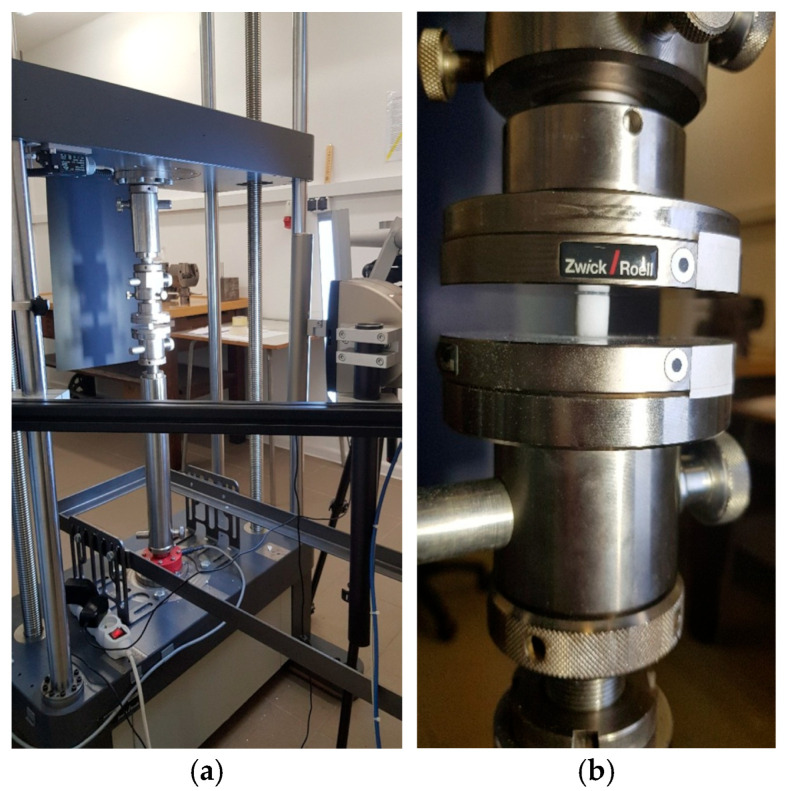
Testing equipment: (**a**) universal testing machine—general view; (**b**) enlarged testing area with sample holders used during compression tests.

The mechanical and microstructural properties of the ceramic samples fabricated from Formlabs Ceramic Resin were examined in two stages, in accordance with the assumptions of the research program. Compression tests were performed using a Zwick/Roell KAPPA DS 50 universal testing machine (Zwick, Ulm, Germany), equipped with an electromechanical drive and a load cell with a capacity of up to 50 kN (Figure 4a,b).

The measurements were carried out at room temperature with a crosshead speed of 2 mm/min. Strain was recorded using a Zwick/Roell video extensometer, which enabled precise determination of the stress–strain curve. Based on the recorded data, the compressive Young’s modulus (E_c_) and compressive strength (σ_c_) were determined. For each combination of printing and burnout parameters, a series of eight measurements was performed, and the obtained results were statistically processed according to Equations (1)–(3) [38].

- Mean value:

(1) $$\bar{x}=\frac{1}{n}\sum_{i=1}^{n}x_{i}$$

- Sample standard deviation:

(2) $$s=\sqrt{\frac{1}{n-1}\sum_{i=1}^{n}{\left(x_{i}-\bar{x}\right)}^{2}}$$

- Two-sided confidence interval:

(3) $$\bar{x}-\frac{t_{0.975,n-1}}{\sqrt{n}}\cdot s<m<\bar{x}+\frac{t_{0.975,n-1}}{\sqrt{n}}\cdot s$$

where $x_{i}$ is the *i*-th measurement value, *n* is the number of samples analyzed, $t_{0.975,\;n-1}$ is the quantile of Student’s t-distribution for a confidence level of 1−α/2 and n−1 degrees of freedom, and *m* is the unknown population mean.

After performing statistical analysis, which allowed the rejection of samples whose values fell outside the calculated confidence interval, a corrected mean value ${\overset{¯}{x}}_{s}$, was determined. This value represents the cleaned dataset and serves as a representative mean for further evaluation.

In the second stage of the study, an additional microstructural analysis was carried out using a Keyence VHX-1000 digital optical microscope (Keyence, Osaka, Japan), enabling observation in the magnification range from 20× to 1000×. Before observation, the samples were sectioned and polished using abrasive papers with grit sizes of 80, 220, 500, 1000, and 2000, followed by polishing with a 5 µm diamond suspension. Observations were performed on cross-sectional areas to assess structural uniformity, pore distribution, and possible interlayer defects formed during the printing or sintering processes.

Porosity evaluation was conducted on the recorded cross-sectional images using the ImageJ software, version 1.54p (National Institutes of Health, Bethesda, MD, USA). Image processing included grayscale conversion, background filtering, binary thresholding with a defined pixel range corresponding to pore regions, and measurement of the pore area fraction relative to the total analyzed image area. The analysis was carried out over the entire sample cross-section to obtain representative results for the full material volume. Based on these data, the surface porosity percentage of the samples was calculated. Additionally, pore size distribution histograms were generated from the binary images, presenting the number of pores within specific size intervals. The histogram analysis enabled the assessment of pore distribution uniformity and comparison of the effects of sintering parameters on the microstructure of individual samples.

## 3. Results and Discussion

The study was divided into three stages, covering the analysis of the influence of technological process parameters on the mechanical properties and microstructure of ceramics fabricated using the stereolithography (SLA) method. In Stage I, the effects of sample geometry, printing layer thickness, and the burnout procedure of the polymer matrix on Young’s modulus (E_c_) and compressive strength (σ_c_) were evaluated, allowing the identification of geometric effects and the role of an individualized thermal schedule. Stage II focused on the influence of sintering temperature and holding time on mechanical properties, which made it possible to determine the range of parameters ensuring stable and repeatable results. In Stage III, a quantitative analysis of porosity and pore size distribution was performed for selected samples sintered at 1250, 1271, and 1300 °C, enabling the correlation of microstructural changes with the obtained E_c_ and σ_c_ values, as well as the identification of mechanisms responsible for differences in material behavior during sintering.

### 3.1. Results of Static Compression Tests

Static compression tests were conducted for two stages of the study, and all data were statistically processed according to the procedure described earlier. The results are presented in the form of summary plots that facilitate the identification of the main trends and relationships between process parameters and material properties. Detailed tabular data, including mean values, standard deviations, and confidence interval lengths, are provided in the Appendix A, Table A1, Table A2, Table A3, Table A4, Table A5, Table A6, Table A7, Table A8, Table A9, Table A10, Table A11, Table A12, Table A13 and Table A14.

**Figure 5:**
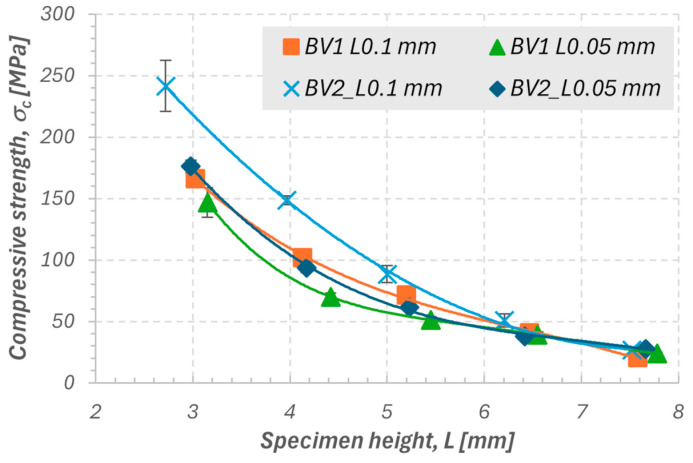
Summary of compressive strength values: BV1 L0.1 mm—first burnout variant with a single layer thickness of 0.1 mm; BV1 L0.05 mm—first burnout variant with a single layer thickness of 0.05 mm; BV2 L0.1 mm—second burnout variant with a single layer thickness of 0.1 mm; BV2 L0.05 mm—second burnout variant with a single layer thickness of 0.05 mm.

**Figure 6:**
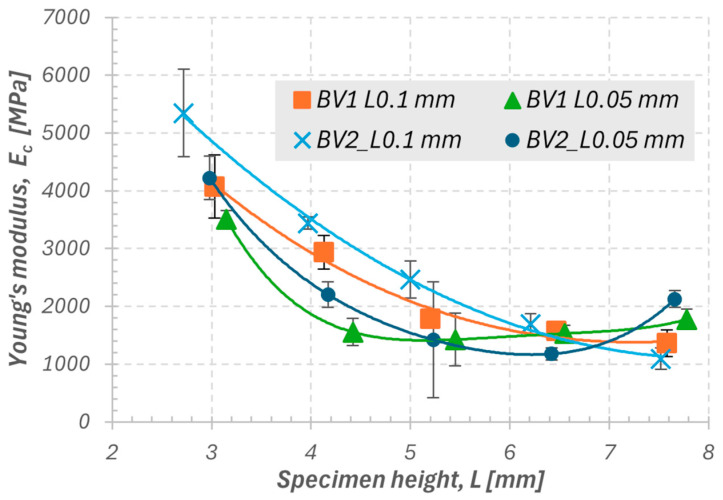
Summary of Young’s modulus values under compression: BV1 L0.1 mm—first burnout variant with a single layer thickness of 0.1 mm; BV1 L0.05 mm—first burnout variant with a single layer thickness of 0.05 mm; BV2 L0.1 mm—second burnout variant with a single layer thickness of 0.1 mm; BV2 L0.05 mm—second burnout variant with a single layer thickness of 0.05 mm.

In the first stage, the effects of sample geometry, printing layer thickness, and the burnout variant of the polymer matrix on the mechanical properties of ceramics fabricated using the stereolithography (SLA) method were analyzed. The results clearly showed that with increasing sample height, compressive strength (σ_c_) systematically decreases (Figure 5), and a similar trend is observed for Young’s modulus (E_c_) in the range of 3 to 6 mm (Figure 6). For greater heights (6–7 mm), E_c_ values no longer show a clear decrease and, in some variants, even slightly increase, which may result from local differences in structure and from a stiffening effect caused by partial surface vitrification or the presence of residual stresses after sintering.

The highest values of both mechanical parameters were obtained for samples produced according to the second heating variant (BV2) with a layer thickness of 0.1 mm. This variant, which applied an individualized burnout time, promoted more complete removal of the polymer matrix and reduction in micropores within the structure, resulting in increased stiffness and strength. The differences between the burnout variants (BV1 and BV2) are less pronounced than those observed between different layer thicknesses. Therefore, the 0.1 mm layer thickness represents the dominant factor determining the material’s ability to carry axial loads. Young’s modulus (E_c_) exhibits greater variability than compressive strength (σ_c_), which corresponds to its higher sensitivity to local structural inhomogeneities, defects in interlayer zones, and variations in the degree of particle bonding. In the samples with the smallest height (approximately 3 mm), the highest E_c_ and σ_c_ values were recorded, accompanied by the largest data scatter. This may result from the difficulty in ensuring uniform densification conditions in small material volumes and the greater influence of surface microstresses and differences in the topography of the printed layers. Conversely, the samples with a height of 4 mm exhibited the smallest scatter of results while maintaining high mechanical parameter values. Therefore, this dimension was selected as representative for further stages of the study, providing a balance between repeatability, uniformity, and appropriate resolution of the printed structure.

**Figure 7:**
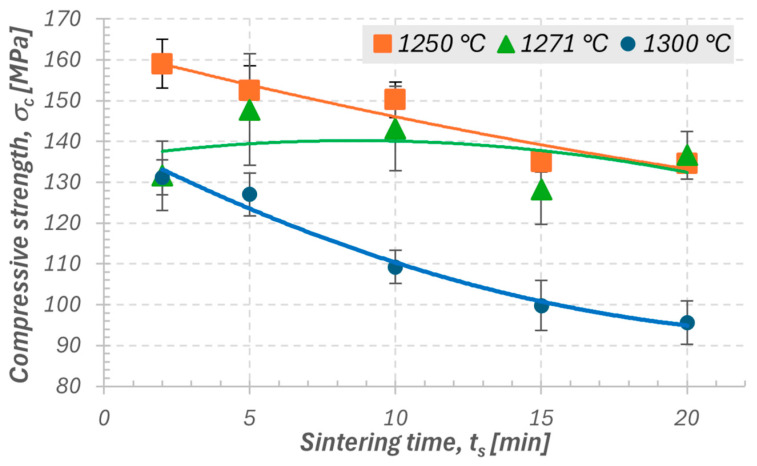
Effect of sintering time of ceramic samples at the three analyzed temperatures on compressive strength.

**Figure 8:**
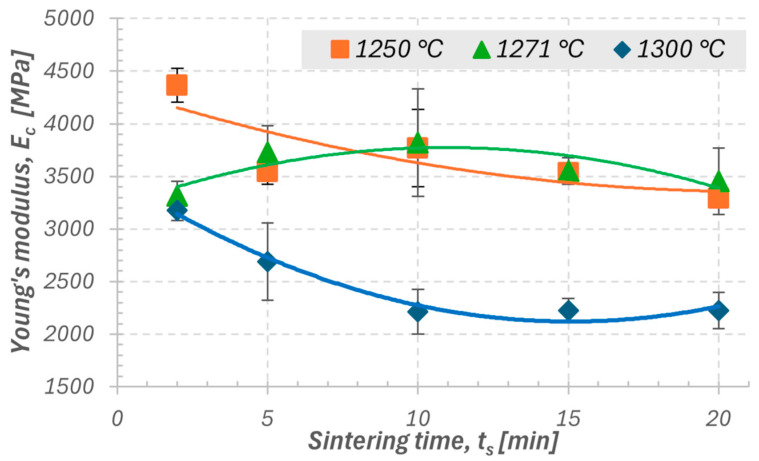
Effect of sintering time of ceramic samples at the three analyzed temperatures on Young’s modulus.

In the second stage of the study, the influence of sintering temperature (1250, 1271, and 1300 °C) and holding time (2–20 min) on the mechanical properties of samples with a height of 4 mm, printed with a layer thickness of 0.10 mm, was analyzed. The results showed that both temperature and sintering time have a significant effect on the mechanical properties of silica ceramics. As shown in Figure 7 and Figure 8, for sintering temperatures of 1250 and 1300 °C, a clear decreasing trend was observed for both compressive strength (σ_c_) and Young’s modulus (E_c_) with increasing holding time. This indicates that prolonged exposure at these temperatures may lead to excessive grain growth or the onset of vitrification, resulting in reduced mechanical strength. For the temperature of 1271 °C, the relationship is not clearly defined. The E_c_ and σ_c_ values do not show a distinct increasing or decreasing trend, which may suggest that at this temperature, the process conditions are close to optimal, and the sintering time has a limited effect on the final properties. The densification process appears to proceed in a stable manner already in the early stages of sintering. The standard deviation values for 1271 °C are slightly higher compared to the other cases, which may result from local microstructural variations, such as inhomogeneities in the distribution of glassy phases or differences in shrinkage during cooling.

For the shortest sintering time (2 min), the standard deviations were the smallest across all analyzed temperatures, confirming the beneficial effect of a short thermal cycle on process stability and repeatability. The most favorable mechanical properties were obtained within the temperature range of 1250–1271 °C and a sintering time of 5–10 min. Under these conditions, the samples exhibited high strength, good stiffness, and low data scatter. At 1300 °C, a small scatter of results was also recorded; however, this was due to the repeatable degradation of the structure. The samples sintered at this temperature underwent similar levels of overheating and vitrification, resulting in comparable but lower mechanical property values.

The analysis of data variability showed that the parameter σ_c_ exhibited lower variation and higher repeatability compared to E_c_, which allows it to be considered the primary indicator of sintering process quality. Young’s modulus, on the other hand, serves as a complementary parameter describing the stiffness and structural uniformity of the material. As a result, it can be concluded that a temperature of 1250 °C with sintering times of 2–10 min favors the maximization of strength while maintaining high process stability, whereas 1271 °C in the range of 5–10 min provides a balance between stiffness, repeatability, and microstructural uniformity. These parameters can therefore be regarded as optimal for achieving stable mechanical properties of ceramics produced by the SLA method.

Furthermore, the results indicate that shortening the sintering time within the optimal temperature range may be beneficial for maintaining high strength and minimizing undesirable thermal effects. In the future, it may be of interest to conduct experiments at temperatures below 1250 °C to determine the minimum conditions ensuring adequate consolidation while preserving high structural integrity.

### 3.2. Results of Microstructural Porosity Analysis

**Figure 9:**
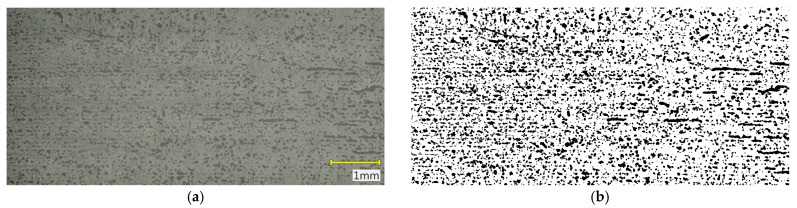
Cross-section of the sample sintered at 1250 °C: (**a**) original color image, (**b**) binary image.

**Figure 10:**
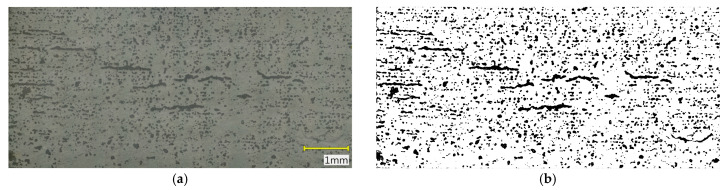
Cross-section of the sample sintered at 1271 °C: (**a**) original color image, (**b**) binary image.

**Figure 11:**
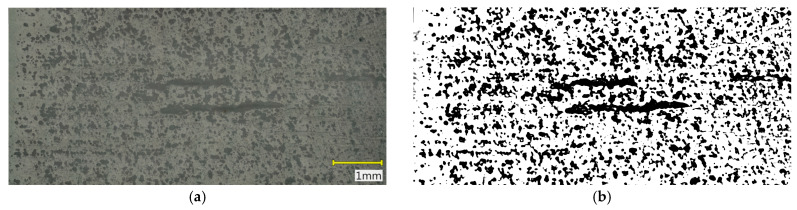
Cross-section of the sample sintered at 1300 °C: (**a**) original color image, (**b**) binary image.

**Figure 12:**
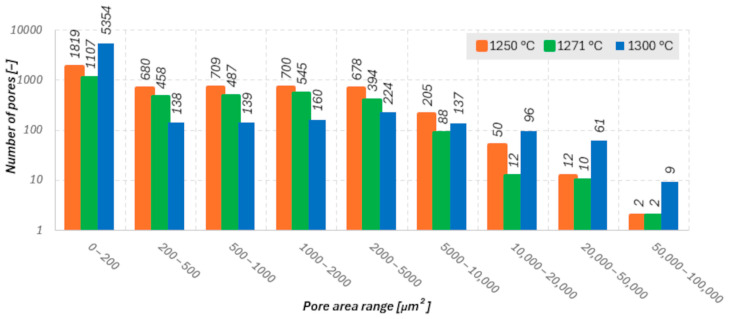
Number of pores within specified size ranges for the three analyzed temperatures.

**Figure 13:**
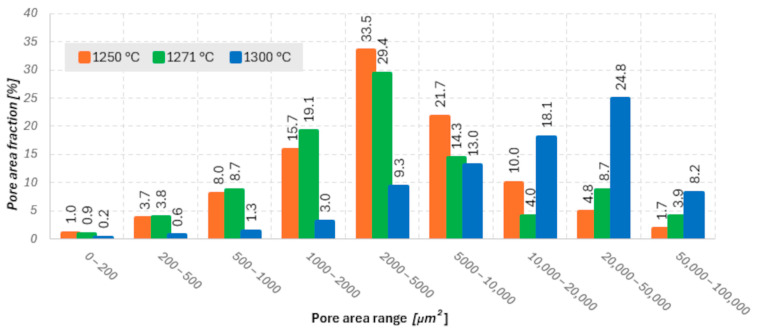
Percentage share of pores within specified surface area ranges for the three analyzed temperatures relative to the total porosity.

Microstructural analysis (Figure 9, Figure 10 and Figure 11) and porosity histograms (Figure 12 and Figure 13) confirm that the sintering temperature significantly affects both the total porosity and the pore size distribution.

The total porosity was 24.4% for 1250 °C, 15.6% for 1271 °C, and 29.1% for 1300 °C, indicating that both excessively low and excessively high sintering temperatures lead to a deterioration of material compactness, caused by incomplete densification in the former case and secondary pore coalescence in the latter.

In all samples, the largest number of pores falls within the range of 0–200 µm^2^, but their contribution to the total porosity remains negligible (<1%). In the range of 200–2000 µm^2^, a gradual decrease in the number of pores is observed with increasing temperature, indicating an effective densification process within this size range (Figure 12). The highest pore area fraction for the samples sintered at 1250 °C and 1271 °C occurs in the range of 2000–5000 µm^2^, suggesting a similar stage of material consolidation (Figure 13).

Although the sample sintered at 1271 °C exhibits the lowest total porosity and a higher Young’s modulus (E_c_), its microstructure is not fully homogeneous. In the central regions of the cross-section, elongated pores oriented parallel to the printing layers are visible, which may act as structural notches and local stress concentrators. In contrast, the sample sintered at 1250 °C shows a more uniform distribution of fine, rounded pores with lower interconnectivity, which promotes better resistance to crack initiation and higher compressive strength (σ_c_). In the case of the sample sintered at 1300 °C, significant structural degradation occurs. The proportion of large pores (>10,000 µm^2^) increases to more than 59% of the total surface area, and the microstructure exhibits interconnected pores and channels with irregular shapes, indicating pore coalescence and partial melting of glassy phases. Such a structure results in a decrease in both σ_c_ and E_c_, along with a large scatter in the values of these parameters.

## 4. Summary and Conclusions

The conducted research confirmed that the mechanical properties and microstructure of ceramics fabricated using the stereolithography (SLA) method strongly depend on the printing parameters, polymer matrix burnout conditions, and sintering parameters. Proper adjustment of the component geometry, printing layer thickness, and individualization of the thermal program allows for obtaining a material with high stiffness, strength, and repeatable mechanical properties of ceramic parts. The results indicate that within the temperature range of 1250–1271 °C, it is possible to achieve a favorable balance between density, structural uniformity, and mechanical strength, which provides a solid foundation for further process optimization. The conclusions are divided into three thematic groups covering the influence of geometry and printing parameters, sintering conditions, and material characterization, followed by a summary of the practical significance of the obtained results.

Influence of geometry and printing parameters:

- Increasing the sample height results in a decrease in Young’s modulus (E_c_) and compressive strength (σ_c_), which is attributed to geometric effects and increasing structural inhomogeneity along the printing axis.
- The highest E_c_ and σ_c_ values were obtained for the second heating variant (BV2) with a layer thickness of 0.10 mm. Layer thickness proved to be the dominant factor determining the quality of interlayer bonding and the efficiency of stress transfer.
- Young’s modulus (E_c_) shows greater data scatter than σ_c_, revealing its higher sensitivity to local inhomogeneities and the degree of particle bonding.
- Samples with a height of 4 mm exhibited the smallest data scatter while maintaining high mechanical parameter values; therefore, they were considered representative for further studies.

Influence of sintering temperature and time:

- For sintering temperatures of 1250 °C and 1300 °C, a clear decreasing trend was observed for both E_c_ and σ_c_ with increasing sintering time, indicating the negative effect of excessive holding on structural stability.
- For the temperature of 1271 °C, the influence of sintering time was ambiguous, suggesting process stability and limited sensitivity of the material to cycle duration within this parameter range.
- The smallest standard deviations were obtained for the shortest sintering time (2 min), confirming the beneficial effect of a short thermal cycle on the repeatability of mechanical properties.
- The most favorable properties (high σ_c_ and E_c_ with low data scatter) were obtained at temperatures of 1250–1271 °C and sintering times of 5–10 min, confirming a stable and efficient thermal process.
- At 1300 °C, despite the small data scatter, a repeatable structural degradation was observed, associated with overheating and partial vitrification, resulting in a decrease in mechanical properties.

Material characterization and general findings:

- The total porosity of samples sintered within the temperature range of 1250–1271 °C falls between 15% and 25%, providing a balance between density and structural uniformity.
- Compressive strength (σ_c_) shows less variability than Young’s modulus (E_c_) and can be considered the main indicator of process quality.
- Further research should include the analysis of lower sintering temperatures (below 1250 °C), which may allow for the reduction in thermal effects while maintaining favorable mechanical properties.

Practical significance:

The obtained results have practical relevance for the optimization of stereolithography-based ceramic manufacturing processes. The relationships identified between sintering temperature, porosity, and mechanical performance provide guidelines for selecting parameters that ensure dense and dimensionally stable structures. These findings confirm the feasibility of producing functional ceramic components using commercial SLA printers, such as the Formlabs Form 2, in combination with experimental ceramic resins. Moreover, the developed methodology can serve as a reference for future studies on new materials, including alumina-based resins recently introduced by Formlabs.

## Figures and Tables

**Table 1:** Summary of the research program.

Research Stage	Scope and Experimental Variables
Stage I—Influence of Geometry and Burnout Parameters (total of 160 samples)	Sample diameter: 10 mm Nominal height: 3–7 mm (in 1 mm increments) Printing layer thickness: 0.05 mm and 0.10 mm Two burnout variants of the polymer matrix: I: constant time, 9 h at 240 °C + 1 h at 300 °C II: time dependent on sample height (5–9 h at 240 °C + 1 h at 300 °C) Sintering: 1271 °C/5 min Compression tests for each parameter combination
Stage II—Influence of Sintering Parameters (total of 120 samples)	Selected geometry: Ø10 × h = 4 mm (layer thickness 0.10 mm) Sintering temperature: 1250, 1271, and 1300 °C Holding time: 2, 5, 10, 15, and 20 min Constant burnout process: 240 °C/6 h + 300 °C/1 h Compression tests for each parameter combination
Stage III—Quantitative analysis of porosity	Samples sintered at 1250, 1271, and 1300 °C (sintering time: 5 min)

## Data Availability

The original contributions presented in this study are included in the article. Further inquiries can be directed to the corresponding author.

## Appendix A

The tables below present a summary of all compression test data subjected to statistical analysis, where

$\bar{x}$—mean value;

s—sample standard deviation;

$x^{-}$—lower confidence interval limit;

$x^{+}$—upper confidence interval limit;

${\bar{x}}_{s}$—corrected mean value;

$s_{s}$—corrected sample standard deviation.

**Table A1:** Statistical analysis of compressive strength (σ_c_) for the first heating variant, single printing layer thickness of 0.1 mm, and sample height L = 3–7 mm.

No	$\sigma_{c,L\approx 3mm}$ [MPa]	$\sigma_{c,L=4mm}$ [MPa]	$\sigma_{c,L=5mm}$ [MPa]	$\sigma_{c,L=6mm}$ [MPa]	$\sigma_{c,L=7mm}$ [MPa]
1	-	-	-	-	41
2	-	101	59	39	25
3	147	100	76	37	19
4	170	108	76	56	19
5	167	103	69	42	16
6	163	96	73	38	29
7	164	102	62	40	16
8	188	111	72	46	18
$\bar{x}\;$	167	103	70	43	23
s	13	5	7	7	9
$x^{-}$	153	98	63	36	16
$x^{+}$	180	108	76	49	30
${\bar{x}}_{s}$	166	102	71	40	20
$s_{s}$	3	1	2	3	5

**Table A2:** Statistical analysis of compressive strength (σ_c_) for the first heating variant, single printing layer thickness of 0.05 mm, and sample height L = 3–7 mm.

No	$\sigma_{c,L\approx 3mm}$ [MPa]	$\sigma_{c,L=4mm}$ [MPa]	$\sigma_{c,L=5mm}$ [MPa]	$\sigma_{c,L=6mm}$ [MPa]	$\sigma_{c,L=7mm}$ [MPa]
1	145	69	57	41	24
2	-	70	52	40	21
3	-	-	54	42	22
4	132	82	51	39	22
5	-	74	52	45	24
6	149	60	43	33	26
7	161	67	45	37	26
8	120	60	47	36	24
$\bar{x}\;$	141	69	50	39	24
s	16	8	5	4	2
$x^{-}$	122	62	46	36	22
$x^{+}$	161	76	54	42	25
${\bar{x}}_{s}$	147	70	51	39	24
$s_{s}$	12	3	3	2	1

**Table A3:** Statistical analysis of compressive strength (σ_c_) for the second heating variant, single printing layer thickness of 0.1 mm, and sample height L = 3–7 mm.

No	$\sigma_{c,L\approx 3mm}$ [MPa]	$\sigma_{c,L=4mm}$ [MPa]	$\sigma_{c,L=5mm}$ [MPa]	$\sigma_{c,L=6mm}$ [MPa]	$\sigma_{c,L=7mm}$ [MPa]
1	306	148	85	55	22
2	241	131	94	75	21
3	270	151	117	51	32
4	237	147	96	59	24
5	229	164	93	49	29
6	236	144	79	47	25
7	236	-	84	44	28
8	197	153	67	40	37
$\bar{x}\;$	244	148	89	53	27
s	32	10	15	11	5
$x^{-}$	217	139	77	43	23
$x^{+}$	271	157	102	62	32
${\bar{x}}_{s}$	242	149	89	51	27
$s_{s}$	21	4	7	5	2

**Table A4:** Statistical analysis of compressive strength (σ_c_) for the second heating variant, single printing layer thickness of 0.05 mm, and sample height L = 3–7 mm.

No	$\sigma_{c,L\approx 3mm}$ [MPa]	$\sigma_{c,L=4mm}$ [MPa]	$\sigma_{c,L=5mm}$ [MPa]	$\sigma_{c,L=6mm}$ [MPa]	$\sigma_{c,L=7mm}$ [MPa]
1	191	85	61	37	23
2	170	97	44	38	25
3	182	101	73	37	31
4	186	85	67	40	24
5	174	99	60	37	27
6	178	95	62	46	31
7	164	83	58	38	25
8	157	89	77	38	35
$\bar{x}\;$	175	92	63	39	28
s	11	7	10	3	4
$x^{-}$	166	86	54	36	24
$x^{+}$	185	98	71	41	31
${\bar{x}}_{s}$	176	94	62	38	28
$s_{s}$	5	4	3	1	3

**Table A5:** Statistical analysis of compressive strength (σ_c_) for sintering at 1250 °C with holding times t_s_ = 2–20 min.

No	$\sigma_{c,ts\approx 2min}$ [MPa]	$\sigma_{c,ts=5min}$ [MPa]	$\sigma_{c,ts=10min}$ [MPa]	$\sigma_{c,ts=15min}$ [MPa]	$\sigma_{c,ts=20min}$ [MPa]
1	181	140	174	141	138
2	164	149	135	137	132
3	161	156	134	128	136
4	144	159	154	145	163
5	149	146	147	134	135
6	145	136	154	129	132
7	159	167	146	132	153
8	162	182	163	137	135
$\bar{x}\;$	158	154	151	135	141
s	12	15	14	6	11
$x^{-}$	148	142	140	131	131
$x^{+}$	168	167	162	140	150
${\bar{x}}_{s}$	159	153	150	135	135
$s_{s}$	6	6	4	2	2

**Table A6:** Statistical analysis of compressive strength (σ_c_) for sintering at 1271 °C with holding times t_s_ = 2–20 min.

No	$\sigma_{c,ts\approx 2min}$ [MPa]	$\sigma_{c,ts=5min}$ [MPa]	$\sigma_{c,ts=10min}$ [MPa]	$\sigma_{c,ts=15min}$ [MPa]	$\sigma_{c,ts=20min}$ [MPa]
1	115	127	103	133	124
2	130	135	149	106	135
3	142	158	159	123	141
4	125	131	148	150	141
5	146	156	146	118	139
6	136	122	154	127	127
7	125	159	126	140	146
8	149	168	136	146	120
$\bar{x}\;$	134	145	140	130	134
s	12	18	18	15	9
$x^{-}$	124	130	125	118	126
$x^{+}$	143	159	155	143	142
${\bar{x}}_{s}$	132	148	143	128	137
$s_{s}$	8	14	10	9	6

**Table A7:** Statistical analysis of compressive strength (σ_c_) for sintering at 1300 °C with holding times t_s_ = 2–20 min.

No	$\sigma_{c,ts\approx 2min}$ [MPa]	$\sigma_{c,ts=5min}$ [MPa]	$\sigma_{c,ts=10min}$ [MPa]	$\sigma_{c,ts=15min}$ [MPa]	$\sigma_{c,ts=20min}$ [MPa]
1	142	101	113	100	100
2	108	102	120	108	95
3	142	133	98	92	72
4	130	129	108	90	100
5	137	131	104	83	114
6	126	103	88	107	68
7	134	135	119	100	96
8	129	121	112	114	87
$\bar{x}\;$	131	119	108	99	92
s	11	15	11	10	15
$x^{-}$	122	107	99	91	79
$x^{+}$	140	132	117	108	104
${\bar{x}}_{s}$	131	127	109	100	96
$s_{s}$	4	5	4	6	5

**Table A8:** Statistical analysis of Young’s modulus (E_c_) for the first heating variant, single printing layer thickness of 0.1 mm, and sample height L = 3–7 mm.

No	$E_{c,L\approx 3mm}$ [MPa]	$E_{c,L=4mm}$ [MPa]	$E_{c,L=5mm}$ [MPa]	$E_{c,L=6mm}$ [MPa]	$E_{c,L=7mm}$ [MPa]
1	-	-	-	-	1421
2	-	3189	1295	1464	1834
3	4709	2247	1732	1029	887
4	3952	3152	1905	2801	1039
5	3577	2420	1669	1505	1986
6	5353	2521	2018	1514	1637
7	4576	2959	1775	1627	1300
8	3550	3109	1819	1754	1092
$\bar{x}\;$	4286	2800	1745	1671	1400
s	715	392	229	547	394
$x^{-}$	3536	2437	1533	1165	1070
$x^{+}$	5037	3163	1957	2176	1729
${\bar{x}}_{s}$	4073	2935	1780	1573	1363
$s_{s}$	546	288	126	118	228

**Table A9:** Statistical analysis of Young’s modulus (E_c_) for the first heating variant, single printing layer thickness of 0.05 mm, and sample height L = 3–7 mm.

No	$E_{c,L\approx 3mm}$ [MPa]	$E_{c,L=4mm}$ [MPa]	$E_{c,L=5mm}$ [MPa]	$E_{c,L=6mm}$ [MPa]	$E_{c,L=7mm}$ [MPa]
1	3631	1108	1120	2269	1919
2	-	2250	1232	1076	1052
3	-	-	1078	1728	1681
4	3505	2168	2100	1618	1978
5	-	1533	2282	2083	1878
6	3588	1071	1677	1425	2081
7	4238	1799	1032	1507	1663
8	3291	1334	1669	1369	1528
$\bar{x}\;$	3651	1609	1524	1634	1723
s	354	480	484	388	327
$x^{-}$	3212	1165	1119	1310	1449
$x^{+}$	4090	2053	1928	1959	1996
${\bar{x}}_{s}$	3504	1555	1425	1529	1775
$s_{s}$	151	233	459	145	176

**Table A10:** Statistical analysis of Young’s modulus (E_c_) for the second heating variant, single printing layer thickness of 0.1 mm, and sample height L = 3–7 mm.

No	$E_{c,L\approx 3mm}$ [MPa]	$E_{c,L=4mm}$ [MPa]	$E_{c,L=5mm}$ [MPa]	$E_{c,L=6mm}$ [MPa]	$E_{c,L=7mm}$ [MPa]
1	6548	3222	2133	1424	373
2	5113	3335	2746	1941	858
3	3931	3216	3041	1644	1177
4	6457	3786	2784	1758	918
5	3483	3960	1609	1392	1506
6	7482	3547	2376	1668	1047
7	5042	-	2369	1385	1225
8	4760	3437	2375	2443	1337
$\bar{x}\;$	5352	3500	2429	1707	1055
s	1373	284	442	356	348
$x^{-}$	4204	3238	2059	1409	764
$x^{+}$	6500	3763	2799	2005	1346
${\bar{x}}_{s}$	5343	3440	2464	1687	1094
$s_{s}$	758	106	317	188	185

**Table A11:** Statistical analysis of Young’s modulus (E_c_) for the second heating variant, single printing layer thickness of 0.05 mm, and sample height L = 3–7 mm.

No	$E_{c,L\approx 3mm}$ [MPa]	$E_{c,L=4mm}$ [MPa]	$E_{c,L=5mm}$ [MPa]	$E_{c,L=6mm}$ [MPa]	$E_{c,L=7mm}$ [MPa]
1	3645	2054	1209	1024	1246
2	4850	2556	392	1071	1953
3	4378	2278	1643	1103	2342
4	4176	885	1977	1238	2053
5	6523	3148	1160	312	1603
6	4449	2904	1417	1246	2087
7	3826	2123	1113	1300	2191
8	3135	2014	3947	1252	2568
$\bar{x}\;$	4373	2245	1607	1068	2005
s	1018	687	1051	321	417
$x^{-}$	3522	1671	729	800	1657
$x^{+}$	5224	2819	2486	1337	2354
${\bar{x}}_{s}$	4221	2205	1420	1176	2125
$s_{s}$	376	221	1003	108	148

**Table A12:** Statistical analysis of Young’s modulus (E_c_) for sintering at 1250 °C with holding times t_s_ = 2–20 min.

No	$E_{c,ts\approx 2min}$ [MPa]	$E_{c,ts=5min}$ [MPa]	$E_{c,ts=10min}$ [MPa]	$E_{c,ts=15min}$ [MPa]	$E_{c,ts=20min}$ [MPa]
1	4468	3084	4299	3823	3294
2	4415	3894	3100	3478	2984
3	4554	3451	3287	3192	3261
4	3667	3679	3434	3507	3362
5	4175	3193	3906	3567	3297
6	4224	3209	4461	3503	3291
7	3330	3505	3781	3081	3529
8	4600	3919	3950	3604	3262
$\bar{x}\;$	4179	3492	3777	3469	3285
s	454	320	479	234	150
$x^{-}$	3800	3224	3377	3274	3160
$x^{+}$	4559	3759	4177	3665	3410
${\bar{x}}_{s}$	4367	3545	3768	3532	3295
$s_{s}$	162	119	368	52	37

**Table A13:** Statistical analysis of Young’s modulus (E_c_) for sintering at 1271 °C with holding times t_s_ = 2–20 min.

No	$E_{c,ts\approx 2min}$ [MPa]	$E_{c,ts=5min}$ [MPa]	$E_{c,ts=10min}$ [MPa]	$E_{c,ts=15min}$ [MPa]	$E_{c,ts=20min}$ [MPa]
1	2829	3360	2385	3159	5129
2	3201	3717	4887	3711	3576
3	3508	3249	3482	3621	3763
4	2914	3251	4241	4485	3048
5	3471	3766	3849	5001	3134
6	3718	3756	3929	3519	3395
7	2848	4798	2994	3528	3795
8	3265	4055	3597	3377	2792
$\bar{x}\;$	3219	3744	3671	3800	3579
s	334	513	761	620	719
$x^{-}$	2940	3315	3034	3281	2978
$x^{+}$	3499	4173	4307	4319	4180
${\bar{x}}_{s}$	3312	3731	3820	3551	3452
$s_{s}$	141	247	511	125	315

**Table A14:** Statistical analysis of Young’s modulus (E_c_) for sintering at 1300 °C with holding times t_s_ = 2–20 min.

No	$E_{c,ts\approx 2min}$ [MPa]	$E_{c,ts=5min}$ [MPa]	$E_{c,ts=10min}$ [MPa]	$E_{c,ts=15min}$ [MPa]	$E_{c,ts=20min}$ [MPa]
1	3395	1751	2383	2375	2384
2	2930	1714	2274	2284	2625
3	2901	3355	2277	2126	1186
4	3190	2852	2057	2099	2251
5	2957	3125	2072	1532	2608
6	3036	2437	1743	2314	1349
7	3264	3681	2621	2141	2039
8	3230	2343	2526	2859	1396
$\bar{x}\;$	3113	2657	2244	2216	1980
s	182	722	282	368	588
$x^{-}$	2961	2054	2008	1909	1488
$x^{+}$	3265	3261	2480	2524	2471
${\bar{x}}_{s}$	3180	2689	2213	2223	2225
$s_{s}$	101	365	213	115	174
